# Synthesis of a new class of carbon–bonded anionic sigma complexes with 1,3-dimethyl-2,6-dioxo-5-(2,4,6-trinitrophenyl)-1,2,3,6-tetrahydropyrimidin-4-olate moiety as insensitive high energy density materials –– implications from impact sensitivity and thermal testings

**DOI:** 10.1186/s13065-014-0078-8

**Published:** 2015-02-01

**Authors:** Rajamani Kulandaiya, Kalaivani Doraisamyraja

**Affiliations:** Post Graduate & Research Department of Chemistry, Seethalakshmi Ramaswami College, Affiliated to Bharathidasan University, Tiruchirappalli, 620 002 Tamil Nadu India

**Keywords:** Insensitive high energy density materials, Molecular salts, 1,3-dimethyl barbituric acid, 2-chloro-1,3,5-trinitrobenzene, Thermal studies, Carbon–bonded anionic sigma complexes

## Abstract

**Background:**

Poly nitro aromatic compounds are high energy density materials. Carbon–bonded anionic sigma complexes derived from them have remarkable thermal stability. At present there is a strong requirement for thermally stable insensitive high energy density materials (IHEDMs) in the energetic field which necessitates the present investigation.

**Results:**

Three new carbon–bonded anionic sigma complexes were synthesized from 2-chloro-1,3,5-trinitrobenzene, 1,3-dimethylpyrimidine-2,4,6(1H,3H,5H)-trione (1,3-dimethylbarbituric acid) and bases such as triethanolamine, pyridine and N,N-diethylaniline, characterized by UV–VIS, IR, ^1^H NMR, ^13^C NMR and elemental analysis data. Their molecular structures were further ascertained through single crystal X-ray diffraction studies. TGA/DTA testings were undertaken at four different heating rates (5, 10, 20 and 40 K/min) and energy of activation was determined employing Ozawa and Kissinger plots.

**Conclusions:**

The reported carbon–bonded anionic sigma complexes were prepared through single pot synthesis in good yield with high purity. These complexes are molecular salts comprise of cation and anion moieties. Because of the salt–like nature, they are highly stable upto 300°C and decompose in two stages on further heating. They are stable towards impact of 2 kg mass hammer upto height limit (160 cm) of the instrument. The delocalization of the negative charge and various hydrogen bonds noticed in their crystals are the added factors of their thermal stability. The new insensitive high energy density materials of the present findings may receive attention in the field of energetics in future.

**Graphical Abstract:**
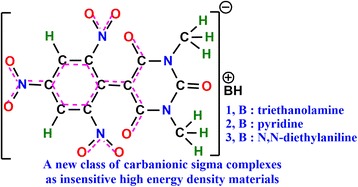
A new class of carbon-bonded anionic sigma complexes as insensitive high energy density materials

## Background

Majority of the aliphatic and aromatic nitro compounds are high energy materials with density greater than ~1.5 g cm^−3^ [1-5]. Nitramines, triazoles, tetrazoles, triazines and tetrazines are heterocyclic nitrogen rich compounds from which several traditional energy rich materials are derived [6,7]. These nitro compounds are vulnerable to explode during storage, transportation and handling due to the sudden impact of mass, friction and heat and hence must be handled scrupulously. In recent years scientists stress on high energy materials which are insensitive towards such factors, eco-friendly cost effective protocol is also insisted by them for wide application in energy production [4,7]. Generally explosives with melting point and decomposition temperatures exceeding 573 K are categorized as thermally stable explosives. Though several thermally stable explosives are reported, their applications are limited in many cases [8-10]. However thermally stable materials with high energy producing performance and with immunity to almost all unplanned stimuli, namely, flame, detonation, high velocity find broad application as propellants, pyrotechnics etc. A widely reported one such insensitive high energy density material is 3-nitro-1,2,4-triazol-5-one (NTO) [11]. 1,1-Diamino-2,2-dinitroethylene (DADNE or FOX-7) has emerged currently due to its superior performance almost comparable to that of RDX and insensitivity characteristics relatively superior to NTO [12,13]. Various nitroimidazole derivatives including 4,5-dinitroimidazole (DNI) and 2,4,5-trinitroimidazole (TNI) have also been investigated as insensitive high energy density materials [14-18]. Ionic energetic molecules possess lower vapour pressure and higher stability than their molecular analogues and hence they are insensitive towards impact and friction [19,20]. Recently hydrazinium 5-aminotetrazolate salt has been identified as insensitive high energy material [6]. Syczewski et al. [21] reported the synthesis of N,N′-dinitro urea (DNU) and its diammonium and dipotassium salts. Though DNU is unstable at room temperature and undergoes decomposition spontaneously, the dipotassium salts are stable at room temperature but start to decompose only after heating to 100°C. A number of impact insensitive dinitromethanide salts have also been reported by He et al. [22]. It has been observed and demonstrated that the presence of organic cations results in relatively insensitive energetic compounds compared to inorganic cations [22]. Pyrimidine salts are also proved as insensitive high energy density materials [23]. As a continuation of our interest in synthesizing insensitive high energy density materials [24] we report in this article a new class of carbon–bonded anionic sigma complexes with 1,3-dimethyl-2,6-dioxo-5-(2,4,6-trinitrophenyl)-1,2,3,6-tetrahydropyrimidin–4–olate moiety synthesized from 2-chloro-1,3,5-trinitrobenzene (TNCB), 1,3-dimethylpyrimidine-2,4,6 (1H,3H,5H)-trione [N,N-dimethylbarbituric acid (NDMBA)] and bases such as triethanolamine (complex 1), pyridine (complex 2) and N,N-diethylaniline (complex 3) as insensitive high energy density materials.

## Experimental

### General

All the chemicals used were of analytical grade. 2-Chloro-1,3,5-trinitrobenzene (TNCB) was synthesized according to the reported procedure from 2,4,6-trinitrophenol (picric acid) and phosphorus oxychloride [25]. The UV–VIS data were recorded on a Shimadzu UV–VIS 1800 spectrophotometer. The IR spectra were obtained using Perkin-Elmer RXI infrared spectrophotometer as KBr pellets. ^1^H NMR and ^13^C NMR spectra were obtained from Bruker DRX-500 MHz spectrometer with (DMSO-d_6_) as solvent and TMS as an internal reference. Good quality diffracting single crystals were obtained by slow evaporation of the solvent at 293 K and mounted on Bruker axs Kappa apex 2 CCD Diffractometer with graphite monochromator. MoK_α_ radiation was used for the measurement. The structure was solved by direct methods and refined by full-matrix least-square method. The non-hydrogen atoms were refined anisotropically. All the hydrogens were placed in their idealized positions and refined as riding on their carrier atoms. The programs used for the crystal-structure determination are – Data collection : APEX2 [26], Cell refinement : APEX2 and SAINT [26]; data reduction : SAINT and XPREP [26]; structure solving : SIR92 [27] ; structure refinement : SHELXL97 [28] ; molecular graphics: ORTEP [29] and Mercury [30]. Instrument (NETZSCHSTA 409C/CD) was used for the TG/DTA studies, at the heating rate of 5 K, 10 K, 20 K and 40 K / min under N_2_(g) purge with alumina powder as reference. The activation energies of exothermic decomposition reactions were determined by Kissinger [31] and Ozawa–Doyle [32,33] methods.

### Preparation of carbon–bonded anionic sigma complex 1

TNCB (2.5 g, 0.01 mol) was dissolved in 40 mL of absolute ethanol and mixed with NDMBA (1.6 g, 0.01 mol) dissolved in 30 mL of absolute ethanol. After mixing these two solutions, 3 mL of triethnaolamine (TEOA; ~0.03 mol) was added, shaken well for 2–3 hours and kept as such at 303 K. After a period of one week, the excess solvent was removed by distillation under reduced pressure during which a pasty mass was obtained. It was washed with 50 mL of dry ether in 5 aliquots when an amorphous solid was obtained. The dry solid was powdered using an agate mortar and once again washed with 30 mL of dry ether and recrystallized from hot ethanol.

### Preparation of carbon–bonded anionic sigma complex 2

TNCB (2.5 g, 0.01 mol) dissolved in 30 mL of absolute ethanol was mixed with NDMBA (1.6 g, 0.01 mol) dissolved in 25 mL of absolute ethanol. To this mixture, 4 mL of pyridine (0.05 mol) was added and shaken well for 2 hours. The solution was filtered and the clear red coloured solution was kept as such at room temperature. After a period of 24 hours, dark shiny maroon red crystalline solid was formed from the solution. The crystalline solid was filtered and washed with 30 mL of dry ether and recrystallized from absolute ethanol.

### Preparation of carbon–bonded anionic sigma complex 3

Equimolar solutions of each of TNCB (2.5 g, 0.01 mol) and NDMBA (1.6 g, 0.01 mol) were prepared in 30 mL of absolute ethanol and mixed well. N,N-diethylaniline (3 mL, ~0.02 mol) was added to this mixture and stirred well using a magnetic stirrer for about 5 hours at 298 K. A dark maroon red coloured pasty mass was obtained after evaporating the solvent. This pasty mass was washed with little amount of hot ethanol and then with 50 mL of dry ether. The dry amorphous solid thus obtained was recrystallized from ethanol. Good quality crystals for single crystal X-ray studies were obtained by slow evaporation of ethanol at room temperature.

### Spectral characterization of complex 1

Maroon red crystals, yield : 75%, IR (KBr) : υ/cm^−1^ ~ 3600-2400 (br), 1681 (s), 1610 (s), 1519 (s), 1336 (s), 677 (s) ; ^1^H NMR (DMSO-d_6_) : cation [C_6_H_16_${\mathrm{NO}}_3^{\oplus }$] δ = 8.76 (br, s, 1H), 5.26 (br, s, 3H), 3.75 (m, 6H), 3.30 (m, 6H); anion, [C_12_H_8_N_5_${\mathrm{O}}_9^{\varTheta }$] δ = 8.60 (s, 2H), 3.08 (s, 6H); ^13^C NMR (DMSO–d_6_); δ = 161.2 (C–8/10), 152.6 (C–9), 149.4 (C–3/5), 141.7 (C–1), 134.6 (C–4), 123.1 (C–2/6), 84.1 (C–7), 55.6 (C–14/16/18), 40.6 (C–13/15/17, overlapped with solvent signal), 27.6 (C–11/12); MS (EI) : m/z (%) 150 (base peak); Micro analysis calcd (%) for C_18_H_26_N_6_O_13_ : C 40.45, H 4.87, N 15.73 ; found (%) : C 41.15, H 4.76, N 16.00; UV/VIS (H_2_O, λ_max_) : 449.5 nm, (EtOH, λ_max_) : 473.5 nm, (DMSO, λ_max_): 508.0 nm.

### Spectral characterization of complex 2

Maroon red crystalline solid, yield : 80%, IR (KBr) : υ/cm^−1^ ~ 3550-2350 (br), 1672 (s), 1584 (s), 1545 (s), 1336 (s) ; 680 (s) ; ^1^H NMR (DMSO-d_6_) : cation [C_5_H_6_ N^⊕^] δ = 8.90 (blurred, d, 2H), 8.53 (t, 1H), 8.02 (t, 2H), anion, [C_12_H_8_N_5_${\mathrm{O}}_9^{\varTheta }$] δ = 8.60 (s, 2H), 3.08 (s, overlapped with solvent, 6H) ; ^13^C NMR (DMSO–d_6_); δ = 161.2 (C–8/10), 152.6 (C–9), 149.4 (C–3/5), 145.6 (C–14), 143.5 (C–16/18), 141.7 (C–1), 134.6 (C–4), 127.3 (C–13/15), 123.1 (C–2/6), 84.1 (C–7); 27.6 (C–11/12); MS(EI) : m/z (%) 80 (base peak) ; Micro analysis calcd (%) for C_17_H_18_N_6_O_11_ : C, 42.32, H 3.73, N 17.43 ; found (%) : C 43.03, H 3.44, N 18.13 ; UV/VIS (H_2_O, λ_max_) : 449.5 nm, (EtOH, λ_max_) : 474.5 nm, (DMSO, λ_max_) : 508.0 nm.

### Spectral characterization of complex 3

Dark maroon red crystals, yield : 86 %, IR (KBr) : υ/cm^−1^ ~ 3550-2300 (br), 1683 (s), 1608 (s), 1521 (s), 1366 (s), 673 (s) ; ^1^H NMR (DMSO-d_6_) : cation [C_10_H_16_ N^⊕^] δ = 10.93 (br, s, 1H), 7.58 (br, s, 5H), 3.56 (br, m, overlapped with solvent, 4H), 1.00 (t, 6H) ; anion, [C_12_H_8_N_5_${\mathrm{O}}_9^{\varTheta }$] δ = 8.60 (s, 2H), 3.08 (s, 6H) ; ^13^C NMR (DMSO–d_6_); δ = 161.2 (C–8/10), 152.6 (C–9), 149.5 (C–3/5), 141.7 (C–1), 134.6 (C–4), 130.7 (C–13-18), 123.0 (C–2/6), 84.1 (C–7), 52.9 (C–19/21), 27.6 (C–11/12); 10.9 (C–20/22); MS (EI) : m/z (%) 150 (base peak); Micro analysis calcd (%) for C_17_H_18_N_6_O_11_ : C 51.16, H 4.65, N 16.28 ; found (%) : C 50.09, H 4.20, N 15.90 ; UV/VIS (H_2_O, λ_max_) : 448.5 nm, (EtOH, λ_max_) : 463.2 nm, (DMSO, λ_max_) : 508.0 nm.

## Results and discussion

**Figure 1:**
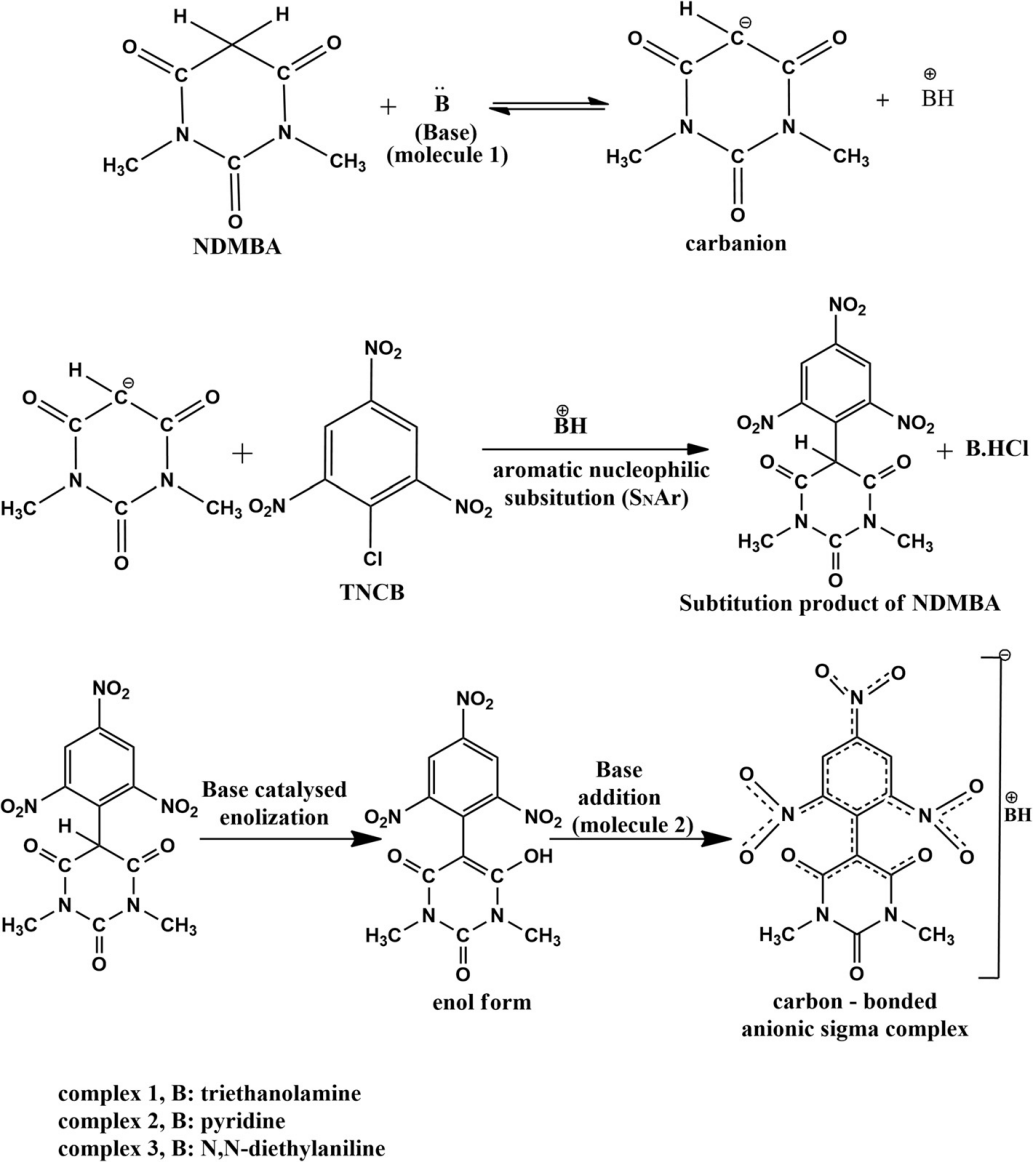
**Schematic representation of the formation of a new class of carbon–bonded anionic sigma complexes 1–3.**

Since the aromatic nitro compound chosen for the present investigation (TNCB) contains good leaving chloro group, carbanion attacks the carbon atom bearing chlorine atom. As nitro groups are present at the ortho and para positions with respect to chloro group, carbanionic sigma complex intermediate is formed followed by elimination of chloro group (SNAr) mechanism, substitution product [5-(2,4,6-trinitrophenyl)-1,3-dimethylbarbituric acid] is formed. Because of the introduction of one more electron–withdrawing group at the 5-carbon of 1,3-dimethyl barbituric acid, the second hydrogen atom of active methylene group of it is made still more acidic which is abstracted by the second molecule of base resulting in the formation of a new class of carbon–bonded anionic sigma complex. This complex comprises of both anion and cation moieties and behaves like a molecular salt. The schematic representation of the formation of a new class of carbon–bonded anionic sigma complexes from the reactants namely 2-chloro-1,3,5-trinitrobenzene, 1,3-dimethylbarbituric acid and bases such as triethanolamine, pyridine and N,N-diethylaniline is presented in Figure 1. IR, ^1^H NMR, ^13^C NMR, UV–VIS, Mass and elemental analysis data strongly support the structure presented here.

In the IR spectrum of TNCB, corresponding to the stretching vibrational mode of C–Cl bond, a strong sharp absorption band is noticed at ~718 cm^−1^, which is absent in the carbon–bonded anionic sigma complexes 1 to 3 clearly denotes that there is removal of chlorine atom during the formation of them. In TNCB sharp strong absorption bands are observed at 1538 and 1342 cm^−1^ and they are assigned to asymmetric and symmetric stretching vibrational modes respectively of N–O bond of the nitro groups. In the IR spectra of the synthesized complexes 1–3 also these bands are noticed at slightly lower frequency regions evidencing the presence of nitroaromatic moiety in them. N–O bond is stretched in the complexes 1–3 due to delocalization of negative charge and hence shift to lower frequency region is understandable. In the IR spectra of all complexes, a broad band which extends from ~2300 to 3500 cm^−1^ is observed, supporting the fact that they are amine salts [34]. Another sharp strong band, characteristic of torsional oscillation of cation moiety (amine salt) [35] is also observed at ~675 cm^−1^ in all complexes 1–3. The stretching vibrational mode of C = C bond appears at lower frequency region (~1600 cm^−1^) substantiating the stretching of this bond due to delocalization of negative charge in the complexes. In the complexes 1–3, the dispersal of negative charge extends upto keto group and accordingly the band with respect to C = O stretching mode appears at a lower frequency region i.e. at ~1680 cm^−1^ than the normal value 1720 cm^−1^. In complex 1, the asymmetric and symmetric stretching frequency bands of C–H bond of –CH_2_ groups appear at 2926 and 2854 cm^−1^ respectively. The position of the bands are slightly at the higher frequency region than the normal value may be due to strengthening of C–H bond which results from protonation at the nitrogen atom of amine upon the formation of the complex. Normally C-H bond of pyridine ring shows absorption band at 3080–3010 cm^−1^ [36] but the respective bond exhibits a band at slightly higher frequency (3102 cm^−1^) supporting the strengthening of this bond due to protonation at the nitrogen atom of pyridine ring in complex 2.

The two ring protons of TNCB are under identical environment and exhibit a singlet in the PMR spectrum at δ 9.20 ppm. In carbon–bonded anionic sigma complexes 1–3, this signal is shifted by 0.6 ppm to upfield. Since the negative charge is delocalised in the nitro aromatic ring of the complex, the protons of it are shielded and hence shifted to upfield. Two N–CH_3_ group protons of 1,3-dimethylbarbituric acid resonate at δ 3.12, whereas in the carbon–bonded anionic sigma complex, the negative charge is delocalised, over a large area upto the keto functions nearer to the N–CH_3_ groups which results in the shielding of N–CH_3_ protons and hence shift towards upfield is noticed. The methylene group protons of 1,3-dimethylbarbituric acid resonate at δ 3.70. This signal is missing in the pmr spectrum of the complexes 1–3 proving that these two protons are replaced during the formation of the complexes. PMR spectrum of triethanolamine comprises of three signals, six protons of methylene groups attached to nitrogen atom resonate as a triplet centred at δ 2.55. The other six protons of methylene groups attached to OH groups yield a multiplet centred at δ 3.41 in the pmr spectrum. The three OH protons exhibit a broad singlet centred at δ 4.33. During the formation of complex 1, protonation of the nitrogen atom of TEOA occurs which experiences deshielding effect on the methylene and OH group protons and hence corresponding protons appear slightly at a lower field, δ 3.30, 3.75 and 5.26 respectively. Proton directly attached to positively charged nitrogen atom is highly deshielded and hence resonates in the low field, δ 8.76, as a broad singlet peak. PMR spectrum of pyridine is loaded with three signals, δ 8.2 (2H, adjacent to nitrogen atom), δ 7.5 (1H, para with respect to nitrogen atom) and δ 6.85 (2H, meta with respect to nitrogen atom). In complex 2, due to protonation at the nitrogen atom of pyridine, the protons are deshielded and shifted to low field : δ 8.90, 8.53 and 8.02 respectively. PMR spectrum of N,N-diethylaniline shows three signals : triplet, 6H of methyl groups at δ 1.2; quartet, 4H of methylene groups at δ 3.4 as a multiplet, 5 ring protons at δ 6.8 – 7.2, whereas in complex 3 they appear at δ 1.0 (triplet), δ 3.56 (broad multiplet) and δ 7.58 (broad singlet) respectively. The low field shifts are due to protonation at the nitrogen atom of N,N–diethylaniline during the formation of the complex. PMR data explicitly prove the presence of trinitrophenyl moiety, 1,3-dimethylbarbituric acid moiety and protonated amine moiety.

Four signals are observed in the ^13^C NMR spectrum of TNCB. The signal with respect to carbon atom bearing chlorine atom appears at δ 125.6. ^13^C NMR spectrum of N,N-dimethylbarbituric acid exhibits four signals, where two signals at δ 166.4 and 152.8 are due to the keto group carbon atoms. Methylene group carbon atom appears at δ 40.2 (overlapped with solvent signal) and the signal at δ 28.2 corresponds to the carbon atoms of the methyl groups attached to nitrogen atoms. The ^13^C NMR spectra of the anion moiety of the complexes 1–3 exhibit eight signals corresponding to eight different carbon environments. The adsorption peak at δ 84.1 is neither noticed in the ^13^C NMR spectrum of TNCB nor in that of 1,3-dimethylbarbituic acid but only in the ^13^C NMR spectra of complexes 1–3 is due to the newly formed carbon environment (C = C) [37] at C-5 carbon atom. C-5 of 1,3-dimethyl barbituric acid is sp^3^ hybridised and is converted to sp^2^ hybridised carbon during complexation and hence the shift from δ 40.2 to δ 84.1 is observed. This shift is significant which explicitly proves the formation of complexes 1–3. As expected the carbon atom of methyl group of N–CH_3_ of 1,3-dimethylbarbituric acid is shifted from δ 28.2 to high field (δ 27.6) upon the formation of the complexes 1–3. In complex 2, corresponding to the cation ring carbon atoms, three signals are noticed at δ 127.3, 143.5 and 145.6 ppm. In complex 3, the methyl and methylene group carbon atoms of N,N-diethylanilinium cation resonate at δ 10.9 and 52.9. All the ring carbon atoms of the cation of complex 3 show absorption peak at δ 130.7. All signals with respect to N,N-diethylanilinium ion are of weak intensity.

Carbon–bonded anionic sigma complexes 1–3 are deeply coloured (maroon red) due to the delocalization of negative charge over a large area. The wavelength of maximum absorption has been noticed to be ~474 nm in ethanol for complexes 1 and 2 and 463 nm for complex 3. However, in DMSO this absorption band is shifted to longer wavelength (~508 nm). Since DMSO is an ionizing solvent, it enhances the charge separation of the complex (molecular salt) which results in red shift [38]. The complex is more solvated and stabilized by DMSO rather than other solvents such as water, ethanol, etc., which is inferred from the higher ε_max_ value in the former solvent. Mass spectral analysis of complex 1–3 confirms the presence of cation moiety. Base peaks corresponding to the cation moiety of complexes 1, 2 and 3 are noticed at m/z 150, 80 and 150 respectively. Calculated percentage of C, H and N based on assigned structures of complex 1–3 coincide with the data observed through experiments.

### Crystal structure of complexes 1–3

Single crystal X-ray diffraction results ascertain the structures revealed through spectral data.

### Complex 1

**Figure 2:**
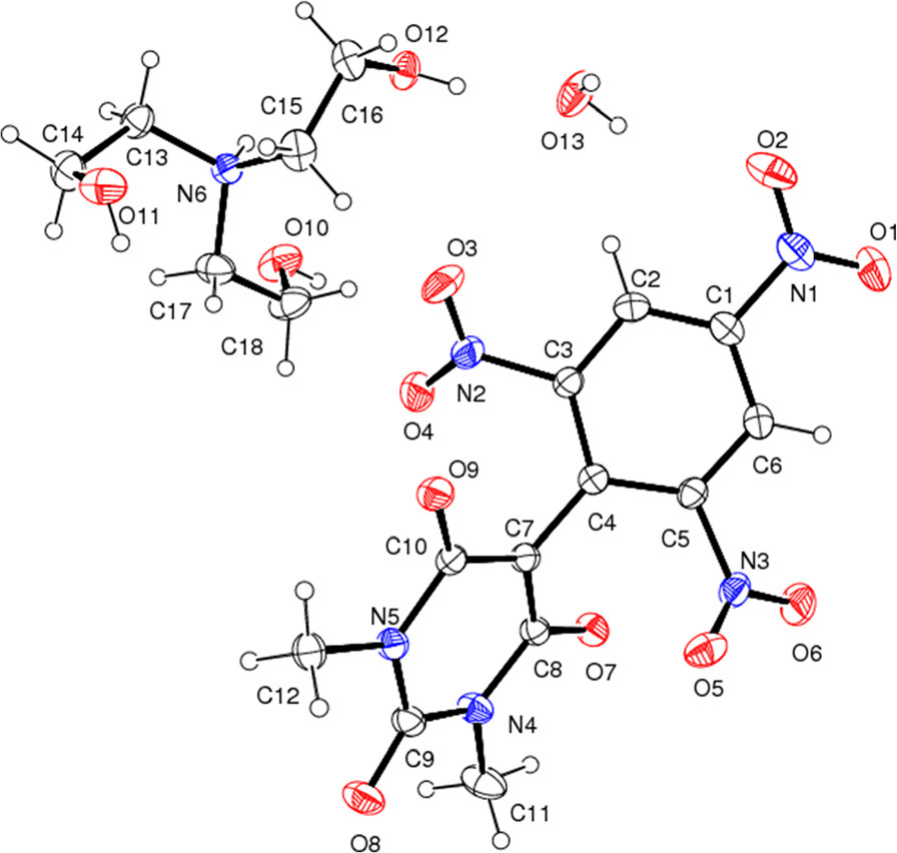
**ORTEP view of carbon–bonded anionic sigma complex 1 showing the atom–numbering scheme.**

**Figure 3:**
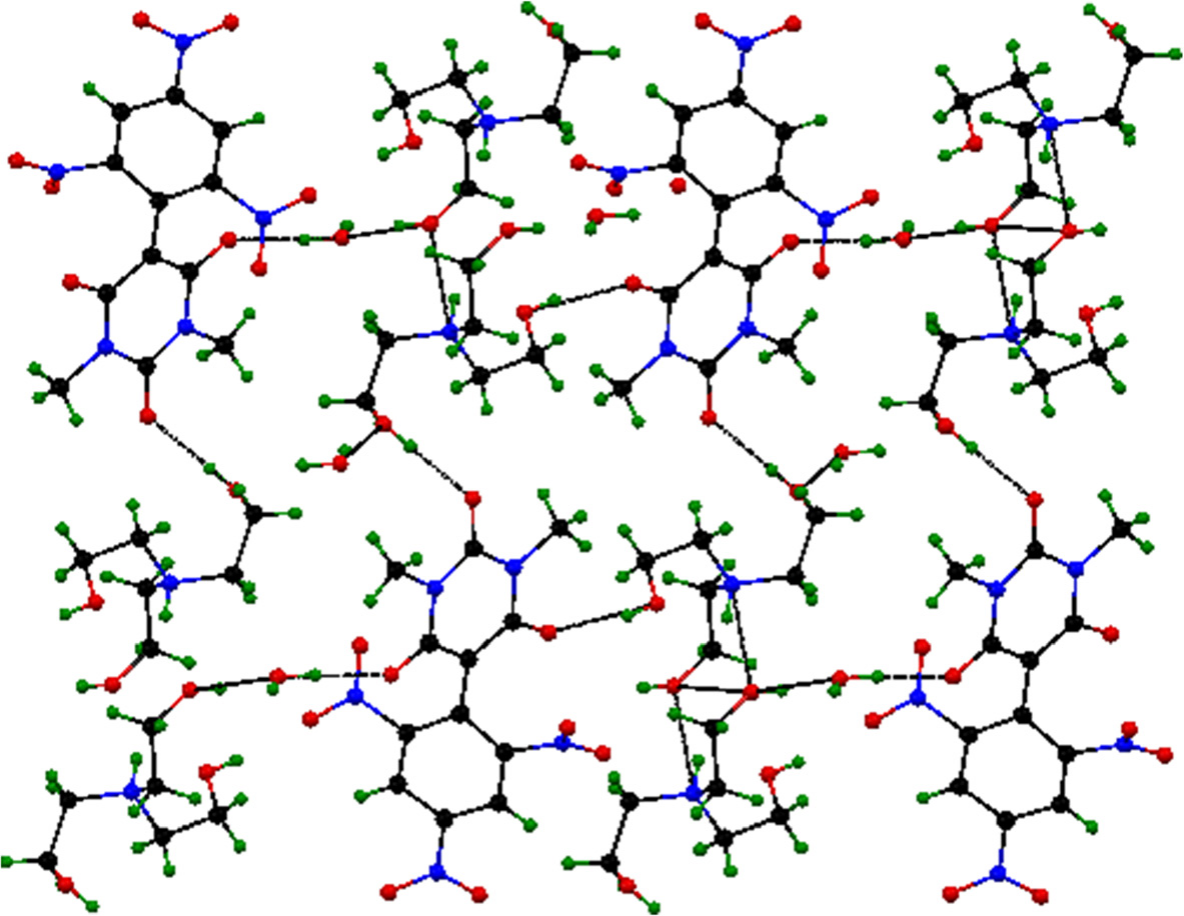
**Crystal packing view of carbon–bonded anionic sigma complex 1.**

(Trivial name : triethanolammonium 5-(2,4,6-trinitrophenyl)-1,3-dimethylbarbiturate) crystallizes in triclinic system with P-1 space group as monohydrate. The important crystallographic data was summarized in Table 1. The asymmetric unit comprises of one cation moiety (C_6_H_16_${\mathrm{NO}}_3^{+}$) one anion moiety (C_12_H_8_N_5_${\mathrm{O}}_9^{-}$) and one molecule of water (Figure 2). Hydroxyl groups (O–H) of water form two strong hydrogen bonds, one with the oxygen atom of the carbonyl function of anion (O7) and other with the oxygen atom of cation (O11). Oxygen atom of water is also linked directly through strong H–bond to the O(12) – H(12) of cation. Thus cation and anion moieties of the carbon–bonded anionic sigma complex 1 are linked through water molecule via H–bonds (Figure 3). O(11) – H(11) group of cation moiety is linked through hydrogen bonding to O(8) of the carbonyl group of anion and O(10) – H(10) of cation moiety is also linked through hydrogen bonding to O(9) of the carbonyl group of anion. Weak N–H…O hydrogen bonding is noticed between N–H group of cation and O(12) of the OH group of cation itself. C(15) – H(15B) of cation is also directly attached to oxygen atom of nitro group of anion [O(3)] through H-bonding. Several other weak C–H…O hydrogen bonds also stabilize the crystal structure (Table 2). It has been observed that the two rings in the anion moiety are not coplanar and twisted by 45.77 (6)°. The nitro group para with respect to the junction of two rings is twisted from the plane of the nitroaromatic ring by 18.09 (17)°. The nitro group [O(3)–N(2)–O(4)] is deviated from the ring to an extend of 45.90 (15)° and the other nitrogroup [O(5)–N(3)–O(6)] is also deviated from the plane of the nitrophenyl ring and the angle of deviation has been observed as 43.24 (9)°. Complex 1 is maroon red in colour due to the delocalization of negative charge on the nitroaromatic moiety. The deviation angle of nitro groups from the plane of the phenyl ring implies that the para nitro group with respect to ring junction [O(1)–N(1)–O(2)] is more involved in the delocalization than the other two nitro groups. It has also been observed that the methyl group attached to N(4) atom of pyrimidine ring is deviated from the plane by an angle 87.67 (4)° and the methyl group attached to N(5) atom of the ring is also deviated [87.70 (4)°]. Thus the methyl groups attached to N(4) and N(5) atoms are almost perpendicular to the plane of pyrimidine ring.

**Table 1:** **Crystallographic data for the carbon–bonded anionic sigma complexes 1 – 3**

**Molecule**	**1**	**2**	**3**
**Empirical formula System, sp. gr** ***., Z***	**C**_**18**_ **H**_**24**_ **N**_**6**_ **O**_**12**_ **Triclinic, P-1, 2**	**C**_**17**_ **H**_**14**_ **N**_**6**_ **O**_**9**_ **Triclinic, P-1, 2**	**C**_**22**_ **H**_**24**_ **N**_**6**_ **O**_**9**_ **Monoclinic, P2**_**1**_ **/n, 4**
*a*, *b*, *c* Å	8.4411(2), 12.1069(4), 13.3113(6)	8.8720(2), 11.2160(4), 11.5280(4)	14.9093(5), 9.4785(3), 17.7118(6)
α, β, γ deg	111.383(2), 101.163(2), 102.748(10)	75.6030(10), 75.0350(10), 90	90, 100.2370(10), 90
*V*, Å^3^	1177.41(8)	1073.40(4)	2463.15(14)
*D*_x_, Mg/m^3^	1.507	1.492	1.393
Radiation, λ, Å	MoKα, 0.71073	MoKα, 0.71073	MoKα, 0.71073
μ, mm^−1^	0.130	0.127	0.110
T, K	293	293	293
Sample size, mm	0.35 *x* 0.30 *x* 0.30	0.20 *x* 0.15 *x* 0.15	0.35 *x* 0.30 *x* 0.30
Diffractometer	Bruker axs kappa apex2 CCD	Bruker axs kappa apex2 CCD	Bruker axs kappa apex2 CCD
Scan mode	ω and φ	ω and φ	ω and φ
Absorption correction	Semi-empirical from equivalents 0.9432,0.9758	Semi-empirical from equivalents 0.9217,0.9965	Semi-empirical from equivalents 0.9536,0.9765
T_min_, T_max_			
θ_max_, deg	25.00	25.00	25.00
*h, k, l* ranges	−12 ≤ h ≤ 11,-15 ≤ k ≤ 15,	−9 ≤ h ≤ 9,-19 ≤ k ≤ 19,	−17 ≤ h ≤ 17,-11 ≤ k ≤ 8,
	-15 ≤ l ≤ 15	−19 ≤ l ≤ 19	−21 ≤ l ≤ 21
No of reflections: measured/unique (*N*1), *R*_int_	20646/4121, 0.0278	18538/3769, 0.0295	21291/4332, 0.0307
Refinement method	Full-matrix least-squares on F2	Full-matrix least-squares on F2	Full-matrix least-squares on F2
No of refined parameters	345	347	338
*R1/wR*2 relative to *N*1	0.0400, 0.1101	0.0386, 0.1041	0.0413, 0.1113
*R*1/wR2 relative to *N*2	0.0459, 0.1167	0.0494, 0.1127	0.0596, 0.1293
*S*	1.041	1.047	1.020
∆ρ_max_/∆ρ_min_, e/Å^3^	0.390, −0.277	0.273, −0.209	0.297,-0.192
Programs	APEX2, SIR92, SHELXL97, ORTEP-3, Mercury	APEX2, SIR92, SHELXL97, ORTEP-3, Mercury	APEX2, SIR92, SHELXL97, ORTEP-3, Mercury

**Table 2:** **Hydrogen bond matrics for carbon–bonded anionic sigma complex 1**

**D-H…A**	**d(D-H)**	**d(H…A)**	**d(D…A)**	**<(DHA)**
C(12)-H(12B)…O(1)#1	0.96	2.62	3.329(3)	130.5
C(14)-H(14A)…O(2)#2	0.97	2.63	3.436(3)	140.4
C(14)-H(14B)…O(2)#3	0.97	2.57	3.495(3)	160.6
C(15)-H(15A)…O(11)	0.97	2.40	3.113(3)	129.7
C(15)-H(15B)…O(3)	0.97	2.54	3.294(3)	134.1
C(17)-H(17A)…O(8)#4	0.97	2.45	3.390(2)	162.5
C(18)-H(18A)…O(6)#5	0.97	2.56	3.397(3)	144.3
O(10)-H(10)…O(9)#6	0.82	2.02	2.8319(18)	171.0
O(11)-H(11)…O(8)#4	0.82	1.93	2.750(2)	178.1
O(12)-H(12)…O(13)	0.82	1.87	2.6689(19)	165.8
O(13)-H(13C)…O(7)#5	0.895(16)	1.827(16)	2.7211(19)	177(3)
O(13)-H(13D)…O(11)#2	0.885(16)	1.953(16)	2.804(2)	161(2)
N(6)-H(6A)…O(12)#7	0.879(15)	1.931(17)	2.7502(19)	154.4(19)

Symmetry transformations used to generate equivalent atoms:

#1 -x,-y + 1,-z.

#2 -x,-y,-z-1.

#3 x,y-1,z.

#4 -x,-y,-z.

#5 -x + 1,-y + 1,-z.

#6 x + 1,y,z.

#7 -x + 1,-y,-z-1.

### Complex 2

**Figure 4:**
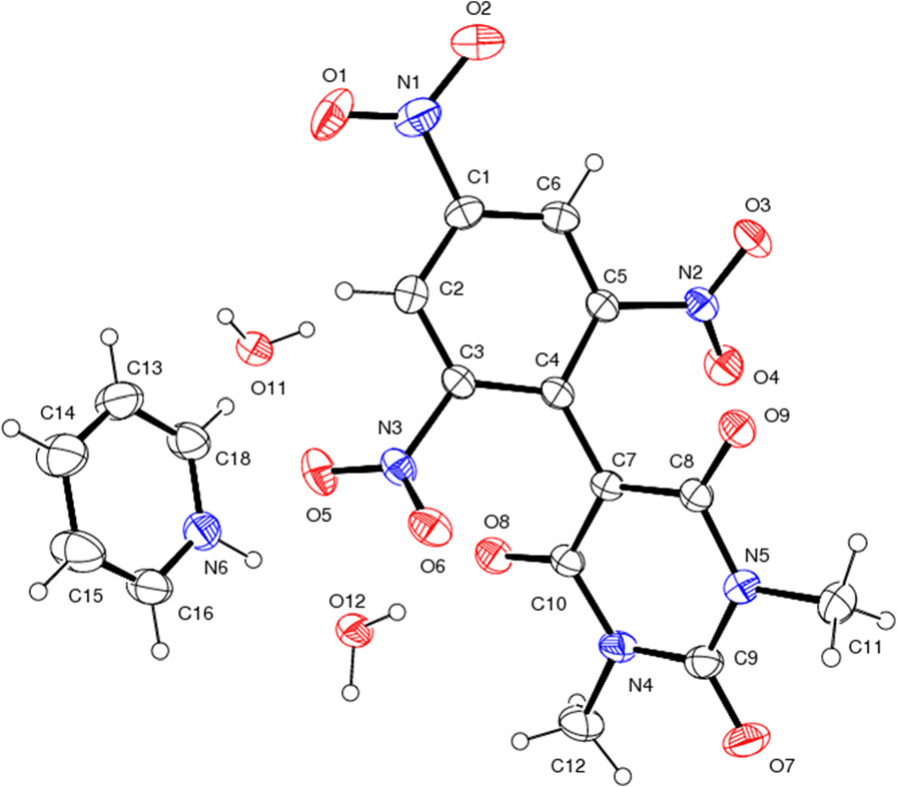
**ORTEP view of carbon–bonded anionic sigma complex 2 showing the atom–numbering scheme.**

**Figure 5:**
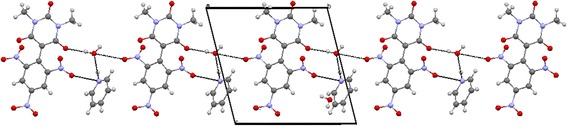
**Crystal packing view of carbon–bonded anionic sigma complex 2.**

Carbon–bonded anionic sigma complex 2 also belongs to triclinic crystal system with P-1 space group. The asymmetric unit is with one cation moiety (C_5_H_6_N^⊕^), one anion moiety (C_12_H_8_N_5_O_9_^−^) and two molecules of water. The ORTEP view of the asymmetric unit is depicted in Figure 4. The two rings in the anion moiety subtend 50.20 (1)°. There may be electrostatic forces of repulsion between the oxygen atoms of carbonyl groups of barbiturate entity and the oxygen atoms of nitro groups of nitroaromatic ring entity which disturbs the coplanarity of the two rings in the anion moiety. As observed in complex 1, the nitro group para with respect to ring junction [O(1)–N(1)–O(2)] bends only slightly from the plane of the benzene ring [11.22 (2)°] and involved more in delocalizing the negative charge than the other two nitro groups [O(3)–N(2)–O(4) ; angle 39.67 (2)°; O(5)–N(3)–O(6); angle 47.37 (1)°]. As observed in complex 1, in complex 2 also the two methyl groups attached to nitrogen atoms of barbiturate unit are nearly perpendicular to the ring [dihedral angle ~ 88^0^]. Interesting hydrogen bonding pattern is observed in the crystal structure of complex 2 (Figure 5). The protonated nitrogen of cationic part forms hydrogen bond with the oxygen atom of one water molecule [N(6)–H(6A) …O(12)] and the O–H group of this water molecule in turn is linked to oxygen atom of the carbonyl group of anionic part through strong hydrogen bond [O(12)–H(12E)…O(8)]. Another O–H group of the same water molecule is hydrogen bonded to oxygen atom of the second water molecule [O(12)–H(12D)…O(11)]. Thus the cation and anion moieties are linked through water molecules via hydrogen bondings which stabilize the crystal system (Table 3). Complex 2 is extraordinarily stable at room temperature may be attributed to these hydrogen bonding network. Hydroxyl groups of second water molecule are also connected to oxygen atoms of carbonyl groups of anion through effective hydrogen bonding [O(11)–H(11D)…O(9) and O(11)–H(11D)…O(7)].

**Table 3:** **Hydrogen bond matrics for carbon–bonded anionic sigma complex 2**

**D-H…A**	**d(D-H)**	**d(H…A)**	**d(D…A)**	**<(DHA)**
O(12)-H(12E)…O(8)	0.86(3)	1.91(3)	2.762(2)	173(3)
O(12)-H(12D)…O(11)#1	0.93(3)	1.85(3)	2.775(2)	176(3)
O(11)-H(11D)…O(9)#2	0.87(4)	1.98(4)	2.841(2)	169(3)
O(11)-H(11E)…O(7)#3	0.90(3)	1.92(3)	2.815(3)	175(3)
N(6)-H(6A)…O(12)	0.94(3)	1.83(3)	2.737(2)	159(3)

Symmetry transformations used to generate equivalent atoms:

#1 -x + 1,-y + 2,-z + 1.

#2 -x + 1,-y + 1,-z + 1.

#3 x,y,z + 1.

### Complex 3

**Figure 6:**
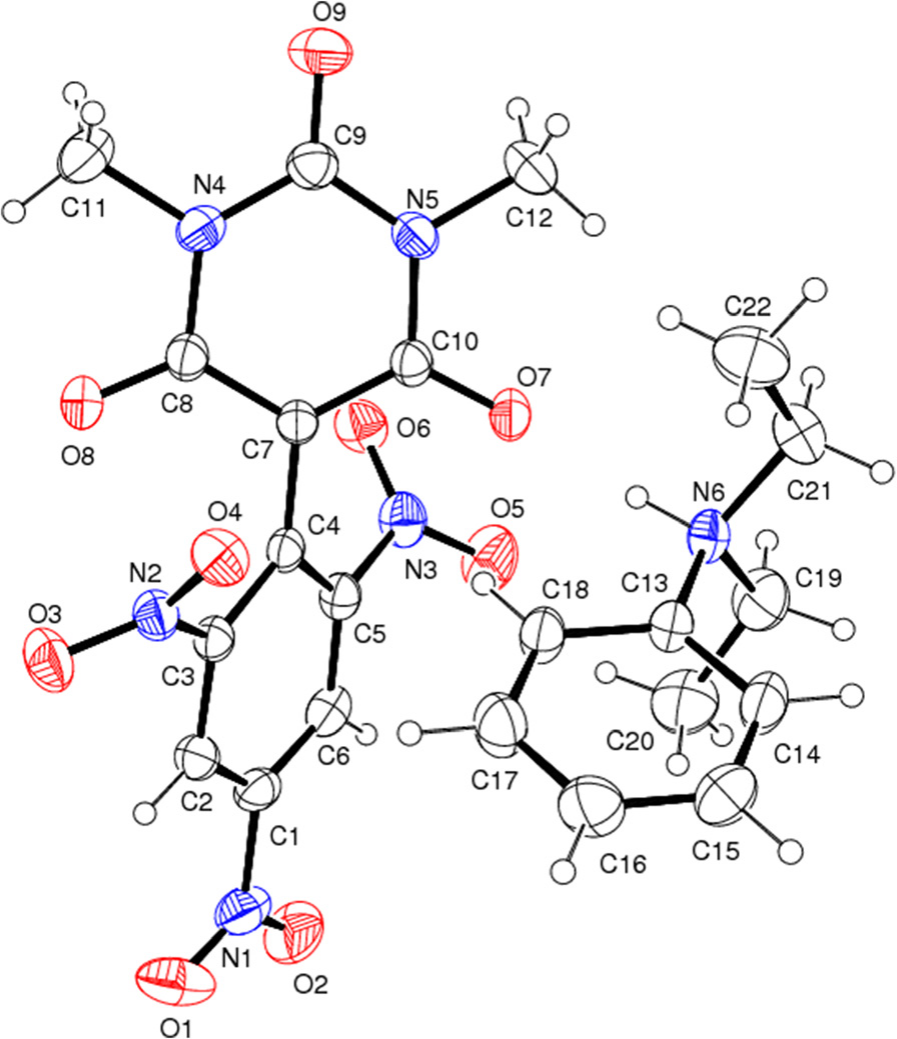
**ORTEP view of carbon–bonded anionic sigma complex 3 showing the atom–numbering scheme.**

**Figure 7:**
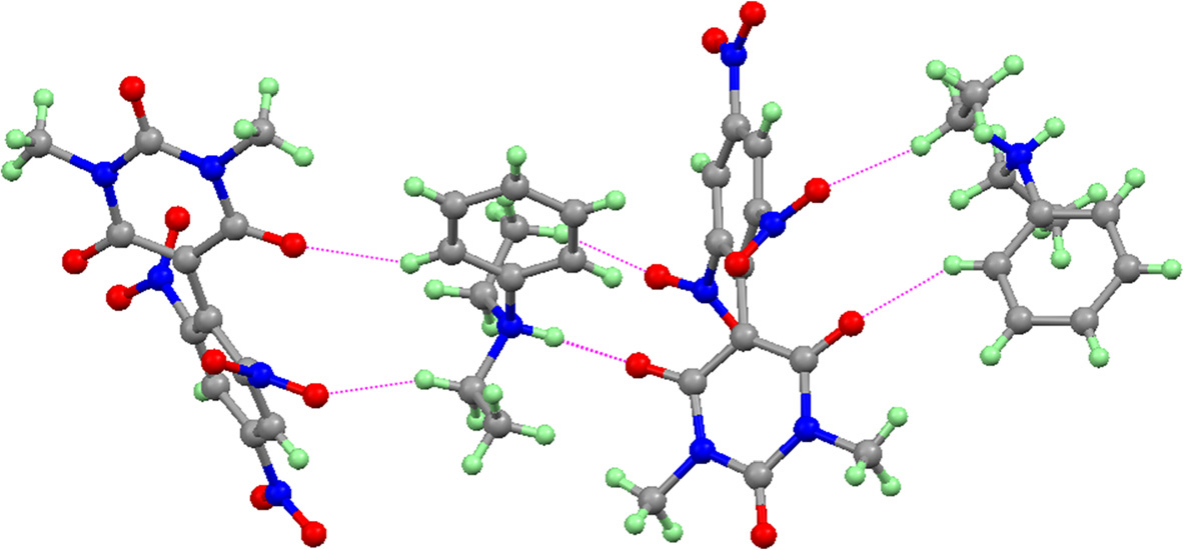
**Crystal packing view of carbon–bonded anionic sigma complex 3.**

Unlike complex 1 & 2, complex 3 crystallizes in the monoclinic system with P2_1_/n space group without water molecule. Figure 6 is the ORTEP diagram of carbon–bonded anionic sigma complex 3 with 30% probability ellipsoid. In complex 3, the rings of the anion are not lying in the same plane and the dihedral angle between their planes is 43.48 (6)°. The nitro group comprises of O(1)–N(1)–O(2) atoms lie almost in the plane of the nitro phenyl ring [1.85 (3)°] and the other two nitro groups are deviating remarkably from the plane of nitro phenyl moiety [O(3)–N(2)–O(4) and O(5)–N(3)–O(6) angles with the plane of the ring with C(1)–C(2)–C(3)–C(4)–C(5)–C(6) atoms, 43.70(4)° and 44.95 (6)° respectively]. The dihedral angle between the plane of methyl groups attached to nitrogen atoms and the plane of barbiturate ring is ~ 90°. The intramolecular hydrogen bond noticed between protonated nitrogen atom of N,N-diethylaniline and the oxygen atom of the carbonyl group [N(6)–H(6A)…O(7)] makes major contribution to the stability of the crystal (Figure 7). Weak hydrogen bond has also been noticed between the C–H group of nitro phenyl ring and the oxygen atom of the carbonyl group of anion moiety and also C–H group of the N,N–diethylaniline ring and the oxygen atom of the carbonyl group of anion moiety. A number of other weak C–H hydrogen bonds formed between the C–H group of methylene and methyl groups of cation and oxygen atoms of the nitro groups of anion moiety also stabilize the crystal (Table 4).

**Table 4:** **Hydrogen bond matrics for carbon–bonded anionic sigma complex 3**

**D-H…A**	**d(D-H)**	**d(H…A)**	**d(D…A)**	**<(DHA)**
C(2)-H(2)…O(9)#1	0.93	2.61	3.074(3)	111.3
C(14)-H(14)…O(8)#2	0.93	2.34	3.171(3)	148.1
C(19)-H(19B)…O(2)#3	0.97	2.31	3.140(3)	143.3
C(20)-H(20B)…O(5)	0.96	2.58	3.413(4)	145.3
C(21)-H(21A)…O(3)#2	0.97	2.52	3.394(3)	149.3
C(21)-H(21B)…O(5)#4	0.97	2.56	3.511(3)	167.4
N(6)-H(6A)…O(7)	0.903(16)	1.847(16)	2.741(2)	170(2)

Symmetry transformations used to generate equivalent atoms:

#1 x,y + 1,z.

#2 x-1/2,-y + 1/2,z-1/2.

#3 -x,-y + 1,-z + 1.

#4 -x,-y,-z + 1.

### Thermal analysis

#### Thermal behaviour of carbon–bonded anionic sigma complexes 1–3

**Figure 8:**
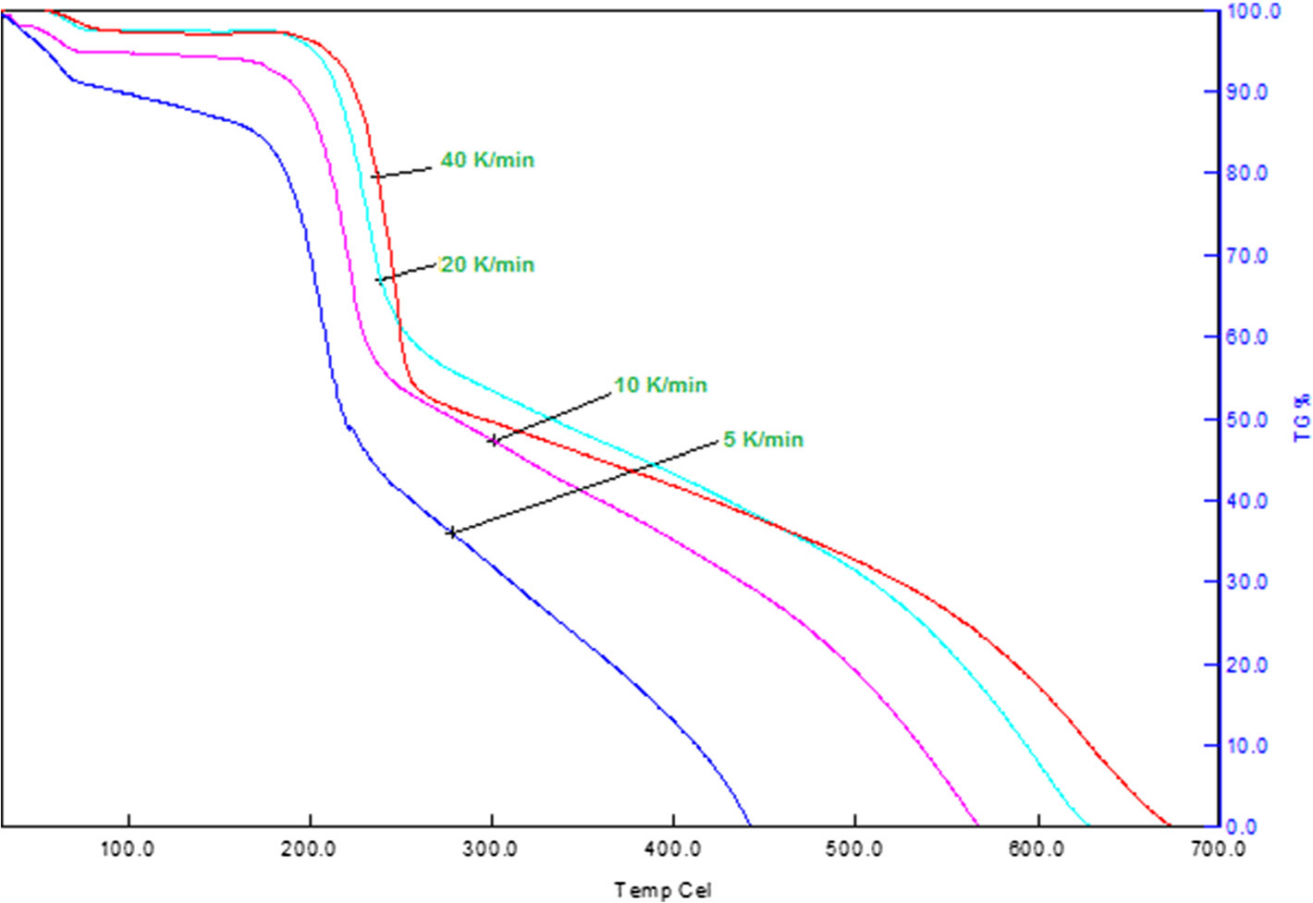
**TGA curves for the decomposition of complex 1 at four different heating rates.**

**Figure 9:**
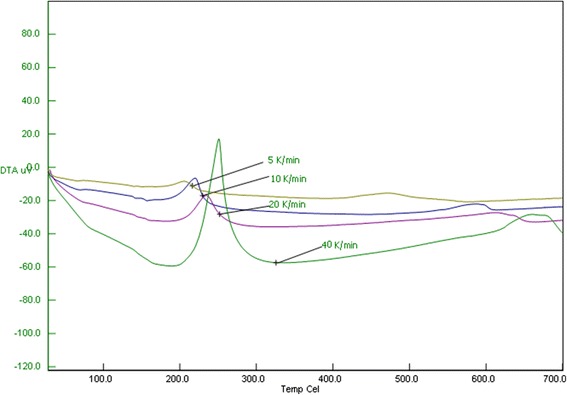
**DTA curves for the decomposition of complex 1 at four different heating rates.**

**Figure 10:**
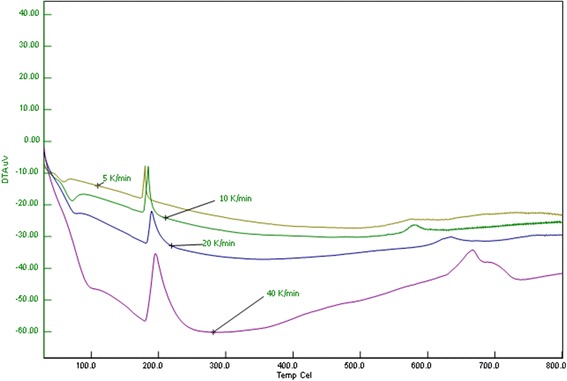
**DTA curves for the decomposition of complex 2 at four different heating rates.**

**Figure 11:**
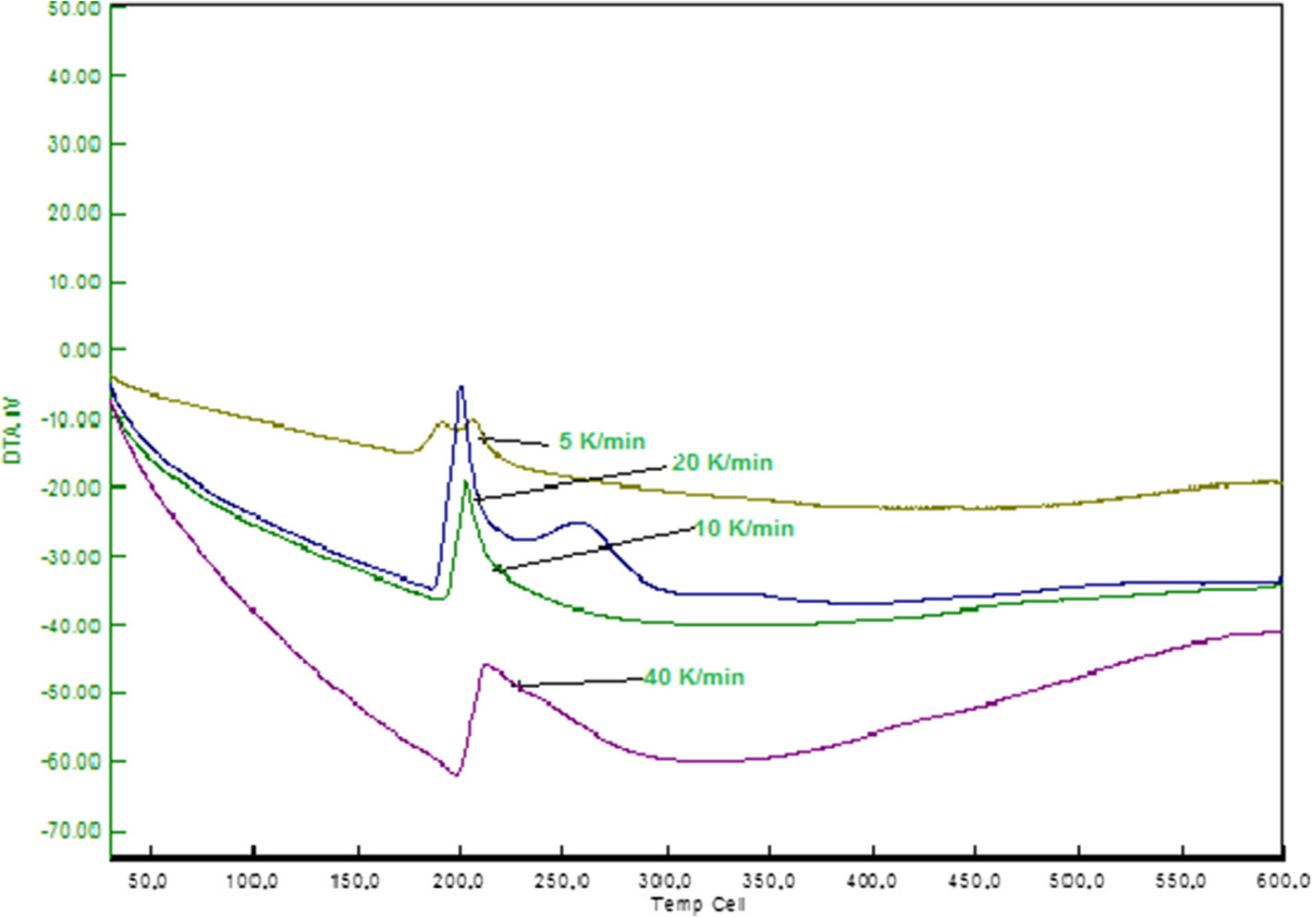
**DTA curves for the decomposition of complex 3 at four different heating rates.**

As TGA/DTA data can determine the thermal stability and decomposition temperatures, the synthesized complexes are subjected to such studies. TGA/DTA curves are generated for the complexes at four different heating rates 5 K, 10 K, 20 K and 40 K/min (Figures 8, 9, 10 and 11). All the three complexes decompose into two stages. Kissinger(eq. 1) [31] and Ozawa–Doyle (eq. 2) [32,33] mathematical relationships are employed to calculate the activation energy.

1 $$ \ln \left(\frac{\upbeta}{{\mathrm{T}}_{\mathrm{p}}^2}\right)= \ln \frac{\mathrm{AR}}{\mathrm{E}}-\frac{\mathrm{E}}{\mathrm{R}}\frac{1}{{\mathrm{T}}_{\mathrm{p}}} $$

2 $$ \log \upbeta +\frac{0.4567\mathrm{E}}{{\mathrm{RT}}_{\mathrm{p}}}=\mathrm{C} $$

where T_p_ is the peak temperature; A is the pre–exponential factor; E is the activation energy; R is the gas constant and B is the heating rate. Log of heating rate versus reciprocal of the absolute temperature is plotted. The slope of the straight line plot is used for the calculation of E. Activation energies for the two stages of the reported complexes are presented in Table 5. The activation energies calculated using the two methods viz Ozawa and Kissinger methods are in good agreement with each other. For complexes 1 and 2 elimination of water has been observed in the heating curves approximately around 100°C. All complexes undergo decomposition after the heating temperature 300°C. Impact sensitivity is determined by fall Hammer apparatus. The synthesized complexes are found to be insensitive towards impact of 2 kg mass hammer upto the height limit (160 cm) of the instrument. Many other energetics materials have also been reported as insensitive towards impact [39,40]. The energy of activation values imply that they are high energy density materials. Impact sensitivity test reveal that they are insensitive materials. The activation energy of complex 3 is higher than that of complex 1. This may be due to the presence of N,N-diethylanilinium aromatic moiety. Similar observation has been reported in the literature [1,7,41]. The activation energy of complex 2 is still higher than complex 3. This may be presumably due to the presence of pyridinium counterpart. It is inferred from the activation energies that the presence of heterocyclic ring increases the thermal stability of materials to a greater extend than the aromatic ring containing only carbon atoms. The synthesized complex 1–3 may be employed safely in commercial applications.

**Table 5:** **The thermal decomposition of carbon – bonded anionic sigma complexes (1 – 3)**

**Complex**	**Stage**	**Activation energy kJ/mol (kcal/mol)**	
		**Using Ozawa method**	**Using Kissinger’s method**
**1**	I	81(19)	80(19)
	II	54(13)	47(11)
**2**	I	225(53)	229(54)
	II	105(24)	100(24)
**3**	I	101(24)	100(24)
	II	156(37)	158(37)

## Conclusions

In summary, 3 new class of carbon – bonded anionic sigma complexes have been synthesized from 2-chloro-1,3,5-trinitrobenzene, 1,3-dimethyl barbituric acid and the bases such as triethanolamine, pyridine and N,N-diethylaniline. Their structure are established and ascertained through spectral and single crystal XRD studies. Impact sensitivity is determined by fall Hammer method and thermal stability and decomposition temperatures are examined through TGA / DTA studies at four different heating rates 5 K, 10 K, 20 K and 40 K/min. Energy of activation has been determined by using Ozawa and Kissinger equations. Sensitivity tests, thermal analysis and activation energy imply that they are insensitive high energy density materials. The reported carbon–bonded anionic sigma complexes are synthesized using ethanol as solvent in good yield (>80%) with high purity as good quality crystals (ethanol poses less environmental problem than other organic solvents). The complexes are non-hygroscopic and extra ordinarily stable at room temperature. Because of the high thermal stability and smooth decomposition attitude, the reported carbon – bonded anionic sigma complexes will receive attention of scientists working in the field of energetics in forthcoming days.

### Supplementary material

CCDC 1008380, 902202 and 989137 contain the supplementary crystallographic data for the complexes 1, 2 and 3 respectively and can be obtained free of charge via http://www.ccdec.cam.ac.uk/conts/retrieving.html or from the Cambridge Crystallographic Data Centre, 12 Union Road, Cambridge CB2IEZ, UK : fax : (+44)1223-336-033; or email : deposit@ccde.cam.ac.uk.
